# Evaluating variations in metabolic profiles during the dry period related to the time of hyperketonemia onset in dairy cows

**DOI:** 10.1371/journal.pone.0289165

**Published:** 2023-08-10

**Authors:** Zelmar Rodriguez, Catalina Picasso-Risso, Tara N. Gaire, Kazuki Nakagawa, Noelle Noyes, Gerard Cramer, Luciano Caixeta

**Affiliations:** 1 Department of Large Animal Clinical Sciences, College of Veterinary Medicine, Michigan State University, East Lansing, MI, United States of America; 2 Department of Veterinary Preventive Medicine, College of Veterinary Medicine, The Ohio State University, Columbus, OH, United States of America; 3 Department of Veterinary Population Medicine, College of Veterinary Medicine, University of Minnesota, Minneapolis, MN, United States of America; 4 Ajinomoto Co., Inc., Kawasaki, Japan; Cornell University College of Veterinary Medicine, UNITED STATES

## Abstract

Hyperketonemia (HYK) in early lactation can have a different impact on health and productivity depending on the timing of HYK onset. While specific metabolites measured during the dry period may serve as biomarkers of HYK, the correlations between metabolites represent a challenge for the use of metabolic profiles dataset, and little has been explored on HYK. This exploratory cohort study aimed a) to characterize the correlations among metabolites measured during the late dry period in dairy cows, and b) to identify biomarkers in the late dry period associated with the onset of HYK at the first (wk1) and second (wk2) week of lactation. Individual blood samples from 440 Holstein dairy cows were collected at 21 ± 3 days before expected parturition. From each sample, 36 different metabolites were measured in serum and plasma. Hyperketonemia was diagnosed in wk1 and wk2 of lactation based on the blood concentration of beta-hydroxybutyrate (BHB > 1.2 mmol/L). Principal component analysis (PCA) was performed to reduce metabolites to a smaller number of uncorrelated components. Multivariable logistic regression models were applied to assess the associations between principal components (PC) and HYK at wk1 only (HYK+ wk1), wk2 only (HYK+ wk2), or both weeks (HYK+ wk1-2). The incidence of HYK was 16.2% in the first week, 13.0% in the second week, and 21.2% within the first two weeks of lactation. The results of PCA highlighted 10 PCs from which two were associated with HYK+ wk1 as compared with cows without HYK during the first two weeks of lactation (non-HYK); the PC a2 led by bilirubin and non-esterified fatty acids (OR = 1.29; 95%CI: 1.02–1.68), and the PC a5 led by alkaline phosphatase (ALP) and gamma-glutamyl transferase (GGT) (OR = 2.77; 95%CI: 1.61–4.97). There was no evidence of an association between any PC and HYK+ wk2 (vs. non-HYK cows). Cows with elevated PC a5 (led by ALP and GGT) in the dry period were 3.18 times more likely to be HYK+ wk1 than HYK+ wk2 (OR: 3.18, 95%CI: 1.34–8.73; *P* = 0.013). Overall, the main hypothesis generated by our exploratory study suggests that cows with biomarkers of liver dysfunction (ALP, GGT, bilirubin) assessed by PCA at 3 weeks before calving are more likely to develop HYK during the first week of lactation compared to the second week. In addition, results suggest that cows with HYK in both of the first two weeks of lactation had an overall metabolic disbalance during the onset of the late dry period, which based on PCs, encompass biomarkers related to glucogenic and ketogenic metabolic pathways as well as liver dysfunction and fatty liver. Further research is needed to determine the underlying mechanisms associated with the different adaptations between cows that develop HYK during the first and second week of lactation.

## Introduction

The transition from late gestation to early lactation is a challenging time for dairy cows [1]. Immune activation and increased inflammation in response to tissue damage, remodeling, and infection [2]; as well as, energy deficit caused by unsatisfied increased demands of nutrients to sustain fetal growth, lactogenesis, and galactopoiesis [3] are hallmarks of this period. This period of negative nutrient balance is characterized by the increased mobilization of body reserves, such as non-esterified fatty acids (NEFA) from adipose tissue, that is metabolized into ketone bodies (i.e., β-hydroxybutyrate [BHB], acetoacetic acid, and acetone) providing an alternative source of energy to peripheral tissues [4]. While this ketogenesis process is considered a fundamental strategy for the adaptation to the cow’s homeorhetic state postpartum [5], an excessive rise of ketone bodies in blood is associated with negative health effects. Hyperketonemia (HYK), defined as elevated concentrations of BHB in blood, has been associated with multiple metabolic and infectious diseases during early lactation [6–8], increased likelihood of herd removal [7, 9, 10], and impaired reproductive performance [10, 11]. In addition, the excessive mobilization of adipose tissue can overwhelm the liver’s capacity to sustain the ketogenesis process leading to the accumulation of triglycerides in the liver and hepatocyte degeneration [5]. As a consequence, liver enzymes (e.g., such as alanine aminotransferase–ALT–, alkaline phosphatase–ALP–, gamma-glutamyl transferase–GGT–) are released into circulation and considered biomarkers of liver dysfunction [12].

The timing when HYK occurs after calving plays a significant role in the negative consequences in affected cows [6, 11]. In a recent study, our group reported that cows that develop HYK within the first week postpartum are more likely to have lower milk production and pregnancy rates, and a higher risk of herd removal than HYK negative cows [13]. However, these differences were not observed when HYK was diagnosed in the second week of lactation. The reasons for the different effects of HYK according to the time of onset are not clear yet, but liver function may play a significant role as the negative nutrient balance around calving not only limits energy availability but also essential amino acids and other metabolites necessary for physiological processes [14]. Hence, the metabolic profile of cows during the dry period can be informative of the liver function and the successful adaptation to the onset of lactation reflected in the HYK status [15]. Consequently, exploring a variety of biomarkers associated with liver dysfunction and metabolic adaptations during the transition period can contribute to our understanding of the reason why HYK occurring at different intervals postpartum may have different consequences on the health and productivity of dairy cows. The value of identifying these biomarkers also has practical implications as it could be used as a tool to target cows at higher risk of developing HYK at different stages of lactation to inform decisions about treatment and prevention strategies. In this exploratory study, we aimed a) to characterize the correlations among metabolites measured three weeks prior to the expected calving date in dairy cows, and b) to identify prepartum biomarkers in the late dry period associated with the onset of HYK at the first (wk1) and second (wk2) week of lactation.

## Materials and methods

### Study population

This exploratory cohort study was conducted on a commercial dairy farm located in Minnesota. The dairy farm had 1,200 lactating Holstein dairy cows (617 cows initially enrolled in the study). Animals were fed a total mixed ration (**TMR**) consisting of 66% forage and 34% concentrate during the dry period, and 60% forage and 40% concentrate for the lactation groups. Both diets contained a standard vitamin and mineral pack, with 18 g of monensin per ton of diet dry matter added to the fresh cow formulation. Pre- and postpartum diets were formulated to meet the nutrient requirement from the NRC for late dry and lactating Holstein cows according to farm conditions [16]. Although different batches of the feed ingredients were used, the diet formulation did not vary during the study period. Cows were housed in free-stall barns with recycled manure solid bedding and headlocks in both dry and fresh cow pens. Lactating cows were milked three times a day, and the farm participated in the Dairy Herd Improvement Association (DHIA) program. After calving, all cows were housed in the same fresh pen until 21 DIM. Multiparous cows received an oral calcium bolus containing 43 g of calcium (Bovikalc, Boehringer Ingelheim) at calving. Standard disease definitions [17] were discussed with farm personnel and herd veterinarian prior to the beginning of the study. Briefly, the standard disease definitions were as follows: clinical mastitis–change in the appearance of the milk or udder, indicative of infection; retained placenta–placenta was considered retained if present (i.e., hanging out of the vulva) for 24 h after calving; milk fever–stage II hypocalcemic parturient paresis based on clinical signs; displaced abomasum–veterinary diagnosis of a right- or left-side abomasal displacement; clinical ketosis–cow that was off feed, having decreased milk production, positive ketone test (over 3.0 mmol/L of BHB), and with no other detectable signs of disease. Farm personnel visually observed postpartum cows daily from 1 to 30 days in milk (DIM) for the clinical signs of diseases. When the disease event was diagnosed, cows received treatment according to farm protocols, and the event was recorded in the farm management software (DairyPlan C21, GEA Farm Technologies). The treatment protocols were based on the herd’s veterinarian recommendation. For clinical ketosis (administered to 2 cows during the study) the treatment included 2 days of intravenous 50% dextrose and 5 days of 300Ml of propylene glycol. The treatment protocol of milk fever was 500Ml of 23% calcium gluconate IV. Clinical mastitis cases received 5 intramammary doses of ceftiofur. Treatment protocols were based on the recommendation by the herd’s veterinarian. The incidence of diseases during the transition period was collected from the farm’s computerized system at the end of the study period.

Throughout the lactation, primiparous and multiparous cows produced on average 36.5 ± 11.2 kg (± SD) and 37.1 ± 5.2 kg/day respectively, had 331 ± 52 lactation days and 55 ± 9 days dry.

All sample activities performed on the farm were approved by the University of Minnesota Institutional Animal Care and Use Committee (1806-36016A).

### Data collection

**Fig 1:**
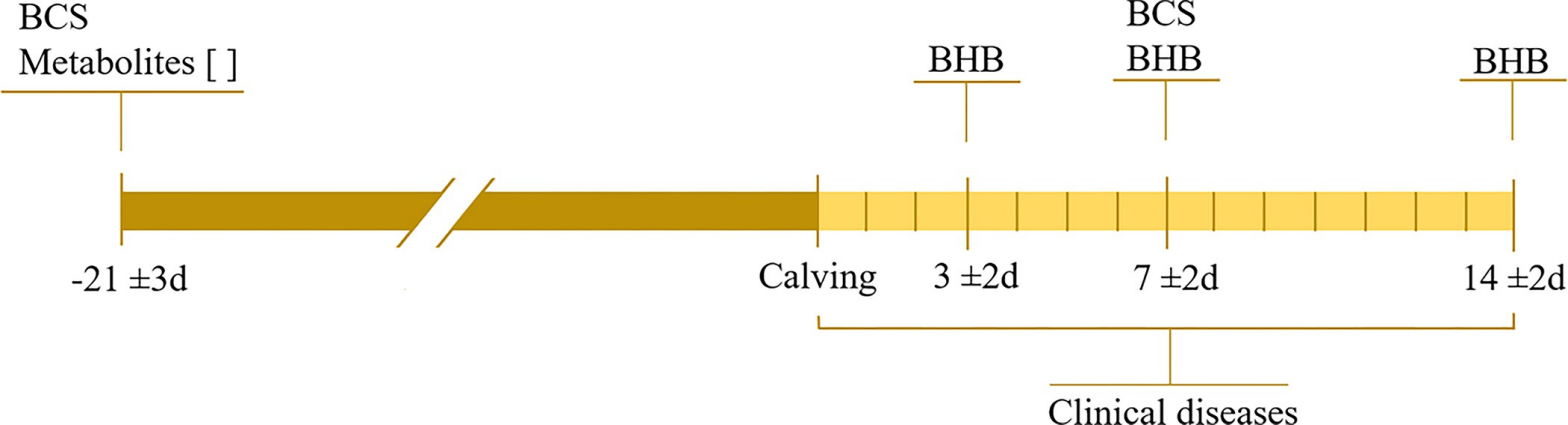
Scheme of blood sampling. Body condition score (BCS) was assessed by researchers. β-hydroxybutyrate (BHB) was measured by researchers and used to determine hyperketonemia status in the first two weeks of lactation. Clinical diseases were collected by farm personnel.

The research team visited the herd three times a week for sampling collection from February to October 2019, enrolling 617 dairy cows both primiparous (defined in this study as those cows starting their first lactation–nulliparous at the time of enrollment) and multiparous (defined as cows starting their second or greater lactation after enrollment). All cows with a pregnancy length of over 250 days were considered eligible for inclusion. Enrollment occurred at 260 ± 3 days of gestation (21 days prior to the estimated calving date). On the day of enrollment, two blood samples were collected immediately after morning feeding (approximately 10:00 am). After parturition, blood samples were collected at days 3 ± 2, 7 ± 2, and 14 ± 2 immediately after the morning milking (approximately 8:00 am) when cows returned to their pen to freshly delivered feed. At enrollment and 7 ± 2 DIM, body condition score (BCS) was assessed using a 5-point scale with 0.25-point increments [18] (Fig 1). The BCS was measured by three researchers trained in the use of the 5-point scale system. Moreover, at the beginning of each visit, the members of the research team rated the first 10 cows together to confirm scoring similarities between observers.

Blood was drained into 10-mL vacuum tubes, one containing Li-heparin and another without anticoagulant (Vacutainer; Becton Dickinson, Franklin Lakes, NJ), and immediately placed in a cooler. All blood samples were collected from the coccygeal vessels using a 20-gauge, 2.54-cm blood collection needle. Within 3 hours of collection, all blood sample tubes were centrifuged at 2,000x *g* for 15 minutes for serum and plasma separation. Plasma and serum aliquots were kept frozen at -80°C until biomarker measurement analysis. Once a week, serum aliquots were shipped to Marshfield Veterinary Labs (Marshfield, Wisconsin) for a standard analysis of blood biochemistry using an Olympus AU5800 analyzer (Beckman Coulter Inc., Brea CA, USA). Biomarkers included albumin (colorimetric method), alkaline phosphatase (ALP; kinetic), alanine aminotransferase (ALT; kinetic), bilirubin (colorimetric), blood urea nitrogen (BUN; enzymatic), calcium (colorimetric), cholesterol (colorimetric), gamma-glutamyl transferase (GGT; enzymatic), globulin (calculation), glucose (enzymatic), non-esterified fatty acids (NEFA; colorimetric), total protein (colorimetric), and triglyceride (colorimetric). The intra-assay coefficients of variation for these biomarkers were under 2.7%. Similarly, one aliquot of plasma was shipped to Ajinomoto Inc. (Tokyo, Japan) for the determination of a full amino-acid profile, which included: alanine, arginine, asparagine, aspartic acid, citrulline, cysteine, glutamine, glutamic acid, glycine, histidine, isoleucine, lysine, leucine, methionine, ornithine, phenylalanine, proline, serine, taurine, threonine, tryptophan, tyrosine, and valine. The amino acids were analyzed using a UPLC method (Waters Acquity UPLC^®^), including a binary solvent manager, a sample manager FL -I, a Tunable UV (TUV) detector, a SQ Detector, and as a reagent a Derivatization Kit (AccQ・Tag^TM^ Ultra). The intra-assay coefficients of variation for these amino acids were under 3.0%.

The postpartum blood BHB concentration was measured using an electronic handheld device, NovaVet (Nova Biomedical Co.) The NovaVet has been previously validated for use in dairy cows with a sensitivity and specificity in blood ranging from 94.9% - 100.0% and 91.8% - 98.3%, respectively [19, 20]. Hyperketonemia was defined as plasma BHB ≥1.2 mmol/L [21, 22]. Thus, cows with at least one BHB measurement ≥1.2 mmol/L during week 1 (day 1 to 7) were considered HYK positive (HYK+) in week 1, otherwise as negative (HYK−). All cows tested in the first week postpartum were also tested in the second week postpartum (day 8 to 14) using the same BHB threshold of 1.2 mmol/L. Thus, HYK status was determined based on one or two samples depending on the actual day of the sample collection. The diagnosis of HYK was based on the BHB results and performed on the first and the second week postpartum independently.

The research personnel did not share the blood BHB measurement with farm personnel and cows were not treated for HYK during the first two weeks of lactation during the study period unless showing clinical signs (i.e., cow off-feed, decreased milk production, depressed attitude; [23]).

### Metabolite grouping

Metabolites were grouped according to similar characteristics such as glucogenic amino acids (n = 17), amino acids intermediates of ketogenic pathways (n = 6), and metabolites related to other pathways (n = 16). Glucogenic amino acids are those that form pyruvic acid or any of the intermediates of the TCA cycle by catabolism and serve as substrates for gluconeogenesis; thus, are used to synthesize glucose in the liver and kidney [24]. Glucogenic amino acids include alanine, glycine, threonine, proline, serine, aspartic acid, asparagine, arginine, histidine, glutamic acid, cysteine, valine, and methionine. Ketogenic amino acids refer to those that form acetoacetic acid or its precursors (i.e., acetyl CoA or acetoacetyl CoA); thus, used to synthesize ketone bodies and fatty acids. Leucine and lysine are exclusively ketogenic amino acids [24, 25]. In addition, isoleucine, tryptophan, tyrosine, and phenylalanine are amino acids intermediates in both the ketogenic and the glucogenic pathways and were included in both groups [24, 26]. Lastly, other blood chemistry analytes and amino acids involved in metabolic functions such as liver function, fat mobilization, or urea cycle, were grouped together. These metabolites included alkaline phosphatase, bilirubin, gamma-glutamyl transferase, taurine, total cholesterol, non-esterified fatty acids, albumin, triglyceride, β-hydroxybutyrate, ornithine, citrulline, blood urea nitrogen, total protein, and calcium. This latter group will be further referred to as “Analytes”.

### Statistical analyses and model-building strategies

Although there are few explicit guidelines for sample size estimation in Principal Component Analysis, two different approaches have been proposed: using a minimum total sample size, or examining the ratio of subjects to variables, as in multiple regression [27]. In terms of minimum total sample size, studies range in their recommendations from an N of 50 to 400 [28]. In terms of ratio of subjects, recommendations range from 5:1 to 10:1, but researchers agree that higher ratios are generally better [29]. The 440 cows included in the analysis (11:1 ratio) after exclusion due to sampling outside intended timeframe (173) or missing laboratory results (5), met both criteria.

Spearman’s correlation coefficient (ρ) was calculated to evaluate the correlation between all the metabolites and represented graphically in a heat map using the functions ‘cor’ and ‘corrplot’ in the R package ‘corrplot’ (version 4.0.2; R Core Team, 2020). Correlation was considered ‘weak’ if ρ was lower than 0.40, ‘moderate’ if ρ was between 0.40 and 0.69, and ‘strong if above 0.70 [30]. The variables in the original data set were analyzed for detection of outlier observations in a graphic form using boxplots. However, there were no deletions because the samples were considered representative without any clear irregular pattern.

Principal component analysis (PCA) was performed to decompose the large number of highly collinear metabolites in the data (likely consequence of superposition of pathways) into a smaller set of uncorrelated constructed latent variables (principal components) which correspond to a linear combination of the original variables using the ‘prcomp’ function in the R package ‘Stats’. Briefly, PCA is a technique for reducing the dimensionality of large data sets while minimizing information loss. It does so by creating new uncorrelated variables (i.e., principal components) that successively maximize variance [31, 32]. For the PCA, the data were centered by subtracting the mean concentration of each metabolite to obtain a mean of 0. Additionally, it was scaled by dividing the rows by their standard deviation to obtain a uniform variance of one to prevent prioritization of variables that are dominant on an absolute scale but not necessarily as relevant [33]. Bartlett’s Sphericity Tests were applied for each of the three groups of metabolites (i.e., glucogenic, ketogenic, and analytes) to statistically compare the observed correlation matrix to the identity matrix and provide evidence regarding the efficiency of the PCA to factorize the original variables. The PCA was performed by group in order to reduce the complexity of the correlation between the variables during the interpretation while controlling for type I error [34–36]. Thus, the metabolites within a group are represented in each of the PCs of the given group.

Principal components were retained based on Horn’s parallel analysis which has been previously validated [37, 38]. An orthogonal Varimax rotation was applied to reduce the correlation between the extracted PC and minimize the number of variables (i.e., analytes and AA) that have high loadings (squared correlations between variables and components) on each PC to facilitate their interpretation. Individual metabolites with a component load ≥0.4 for a given PCA-derived component are reported as composing that component (i.e., dominant variables according to correlation). Scoring coefficients were constructed and used to calculate component scores for each individual cow (weighted sum of the standardized metabolites within that component, weighted on the component loading for each metabolite).

The interpretation of the PC is based on the strength of the correlation of each variable (i.e., metabolites) with each component in either direction. For instance, if one PC is reported to be moderate or strongly positively correlated with one of the original metabolites, it suggests that the component increases with the increase of the given metabolite. Therefore, the PC can be viewed primarily as a measure of the concentration of serine. Sometimes PC may be strongly correlated with more than one of the original variables, suggesting that the PC is mainly a measure of all those strongly correlated metabolites. The correlation between the metabolites and PC is reported in the results section.

The PC identified in each group of metabolites by PCA was used as the explanatory variables to assess their association with HYK status at different intervals. The metabolic factors were compared between a) HYK+ cows only in wk1 with HYK- cows in the first two weeks of lactation (HYK+ wk1 vs. non-HYK); b) HYK+ cows only in wk2 with HYK- cows in the first two weeks of lactation (HYK wk2 vs. non-HYK); 3) HYK+ cows only in wk1 with HYK+ cows only in wk2 (HYK wk1 vs. HYK wk2); and lastly, 4) HYK+ cows in wk1 and wk2 with HYK- cows in the first two weeks of lactation (HYK wk1-2 vs. non-HYK). For these associations, logistic regression models were built for each separate group of metabolites (glucogenic, ketogenic, or analytes) using the ‘glm’ function in the R package ‘lme4’ [39]. Only cows with complete BHB results in both weeks were used in the regression analyses to reduce potential selection bias. Firstly, univariable models were performed for each PC. In the multivariable models all PC’s in the univariate analysis with *P* ≤ 0.20, as well as parity and their interaction terms, days before calving, BCS score at enrollment, and calving season. Days before calving was dichotomized as “≥ 21 to calving” for days -24 to -21, and “< 21 to calving” for days -20 to -18. The BCS score at enrollment was categorized as low if ≤ 3.25, mid if 3.25 to 3.75, or high if ≥ 4.0. Calving season was categorized as Spring if calved from February to April; Summer from May to July and Fall from August and October. Parity was the only variable forced into all final models independently of significance. Retention in the final models was based on likelihood ratio tests following a backward elimination process: variables that significantly improved the –2 log likelihood when comparing a model with and without the covariate of interest (P ≤ 0.05) were retained. Adjusted odds ratios (OR) and respective 95% confidence intervals (95%CI) were used to report the association between the explanatory variable and outcomes.

## Results

### Descriptive statistics

A total of 440 Holstein dairy cows remained in the study sample for the analysis of the correlations between metabolites after the exclusion of 172 cows due to sampling outside the timeframe of 21 ± 3 days before calving and 5 cows due to missing laboratory results. The BHB concentration was measured in 438 cows (99.5%) during the first week of lactation and 384 cows (87.3%) during the second week.

The incidence of HYK was 16.2% (71/438) and 13.0% (50/384) in the first and second week of lactation, respectively, and 21.2% (81/382) within the first two weeks of lactation. First lactation cows represented 40% (n = 176) of the cows while 26% (n = 114) were starting their second lactation and 34% (n = 150) were in their third or greater lactation. Moreover, because status was determined based on one or two samples depending on the actual day of the sample collection, in week 1, 211 cows were sampled once (14.2% HYK) and 227 cows were sampled twice (18.0% HYK). In week 2, 297 cows were sampled once (13.8% HYK), and 87 cows were sampled twice (11.4% HYK). A total of 382 cows (86.8%) had complete BHB results in both weeks from which 31 cows were HYK+ wk1, 26 were HYK+ wk2, 24 were HYK+ wk1-2, and 301 were non-HYK.

At enrollment, 9.5% of the cows had low BCS, 60.0% mid, and 30.5% high with a median BCS of 4.0 (IQR = 3.7–4.2). At 7 ± 2 d post-calving, 11.9%, 59.9%, and 28.2% of the cows were in the low (i.e., ≤ 3.25), mid (i.e., 3.5 and 3.75), or high (i.e., ≥ 4.0) and high BCS categories respectively, with a median BCS of 3.7 ± 0.2. The milk yield, fat, and protein measured at the first DHIA test for primiparous cows were 28.9 kg/d, 4.2%, and 3.1% respectively. In the multiparous cows, milk yield, fat and protein were 34.8 kg/d, 5.0%, and 3.3% respectively. Diseases during the first two weeks of lactation, obtained from the management software, included left displaced abomasum (n = 3), retained placenta (n = 10), clinical mastitis (n = 7), clinical ketosis (n = 2), and milk fiver (n = 3). Because the incidence of each disease was low (less than 3%), we were unable to assess their effect on our results. Therefore, information about disease information was not included in the final model.

### Principle component analysis

**Fig 2:**
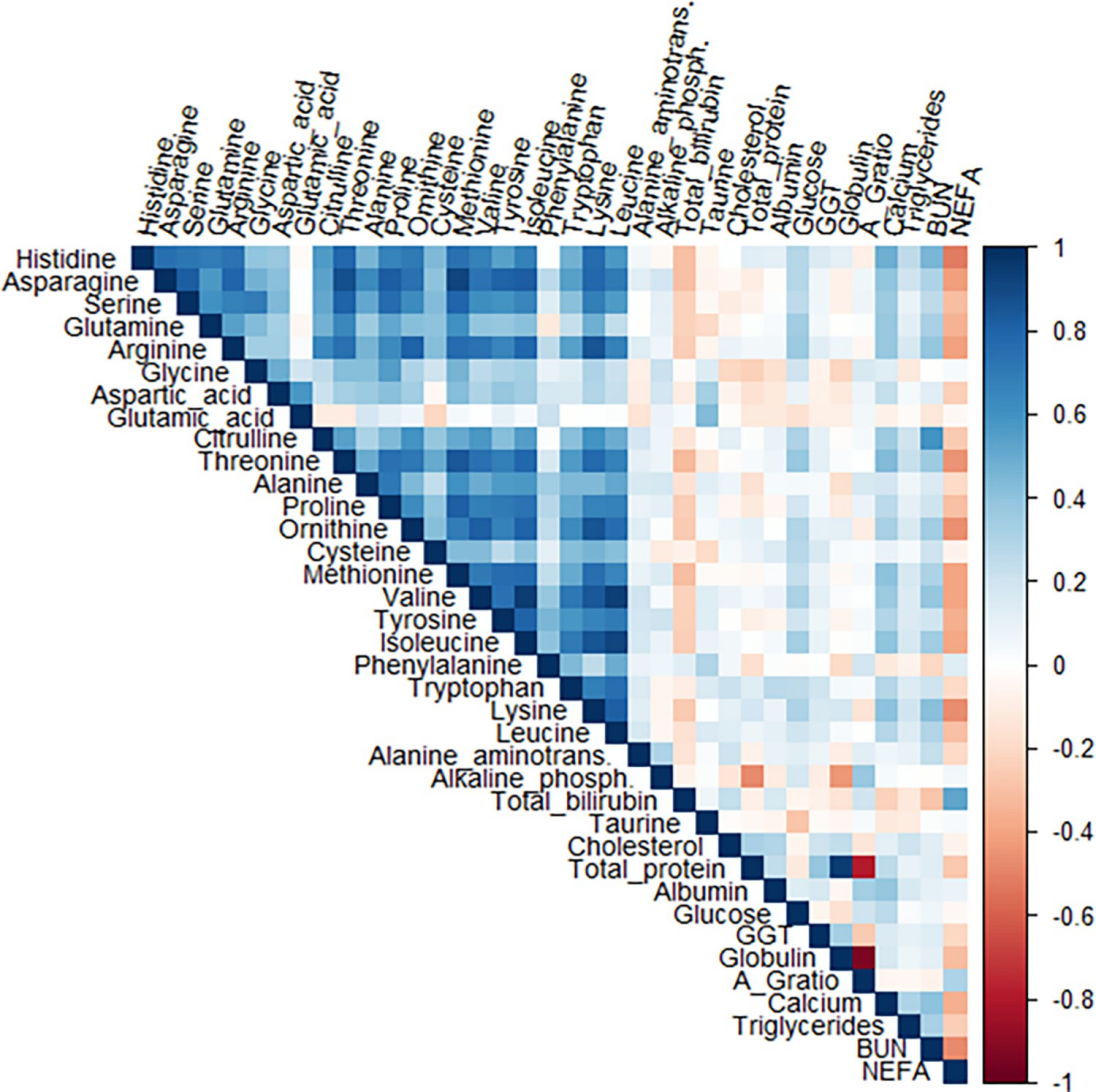
Heat map showing the Spearman correlation matrix for metabolites measured at 21 days before calving date in 440 primiparous and multiparous Holstein dairy cows. The color corresponds to the strength of correlations (blue: positive correlation; white: no correlation; red: negative correlation).

The Bartlett’s Sphericity Tests on the three groups of metabolites (P < 0.001) revealed that the collected data was appropriate to be analyzed by PCA. There were mainly moderate and strong correlations among AA, except for glutamate, taurine, and cysteine which had a positive but weaker correlation with all the other AA. Additionally, most of the amino acids were strongly negatively correlated with NEFA and to a lesser degree with bilirubin. The correlation among blood chemistry analytes was mainly weak (Fig 2).

Based on Horn’s parallel analysis technique, the PCA generated 10 PCs (three among the glucogenic group, one ketogenic, and six among the blood chemistry analytes group). The contribution of each biomarker to each PC is presented in Table 1. The first principal component within the glucogenic pathway (i.e., g1) had the largest eigenvalue (λ) 9.4 and explained about 54% of the variation present within the glucogenic pathway group. Furthermore, the first three PC’s within the glucogenic pathway group (i.e., g1, g2, and g3) explained 74% of the cumulative variation of the group. The PC k1 comprises ketogenic pathway metabolites and contributed 71% of the variance in the ketogenic group. Similarly, PC’s a1 to a6 explained 63% of the variance among the analytes group (Table 2).

**Table 1:** Component loadings indicating the correlation of each metabolite with the principal components (PC) evaluated in principal component analysis of metabolites measured at 21 days prepartum in 440 Holstein dairy cows.

Glucogenic amino acid				Ketogenic amino acid		Blood chemistry analytes						
	g1	g2	g3		k1		a1	a2	a3	a4	a5	a6
Alanine	0.15	0.20	-0.13	Isoleucine	0.48*	Albumin	0.00	0.17	-0.07	-0.19	-0.07	0.50*
Arginine	0.22	-0.05	-0.16	Leucine	0.46*	ALP	-0.06	-0.08	-0.21	-0.12	0.64*	-0.03
Asparagine	0.29	0.02	-0.12	Lysine	0.49*	ALT	-0.12	-0.24	-0.19	0.13	-0.26	0.32
Aspartic ac.	0.13	0.55*	0.08	Phenyl.	0.09	A/G ratio	-0.01	0.15	-0.61*	-0.07	-0.19	-0.01
Cysteine	0.21	-0.28	-0.03	Tryptophan	0.40*	Bilirubin	0.01	0.64*	0.00	0.06	0.04	0.10
Glutamic ac.	-0.11	0.63*	-0.04	Tyrosine	0.38	BUN	0.41*	-0.16	-0.06	0.13	0.05	0.18
Glycine	0.35	0.27	0.28	–	–	Calcium	0.09	-0.04	0.26	-0.30	-0.10	0.36
Histidine	0.35	-0.08	0.03	–	–	Cholest.	0.03	0.15	0.16	0.19	0.06	0.52*
Isoleucine	0.13	-0.02	-0.34	–	–	Citrulline	0.64*	0.08	-0.06	-0.05	-0.01	-0.01
Methionine	0.30	0.03	-0.10	–	–	GGT	0.01	0.05	0.42*	0.13	0.59*	-0.02
Phenyl.	-0.24	0.21	-0.52*	–	–	Glucose	0.18	0.09	-0.02	-0.60*	-0.18	-0.02
Proline	0.28	0.11	-0.08	–	–	NEFA	-0.09	0.59*	-0.07	-0.03	0.01	-0.02
Serine	0.40*	0.04	0.10	–	–	Ornithine	0.55*	-0.05	0.04	-0.06	0.03	-0.10
Threonine	0.33	-0.11	-0.08	–	–	Taurine	0.22	0.13	-0.04	0.63*	-0.24	0.00
Tryptophan	0.00	-0.08	-0.44*	–	–	T.Protein	-0.07	0.04	0.48*	-0.08	-0.06	-0.02
Tyrosine	0.08	0.09	-0.34	–	–	TAG	-0.05	-0.21	-0.14	0.04	0.17	0.43
Valine	0.11	-0.06	-0.36	–	–	–	–	–	–	–	–	–

Metabolite abbreviations: Phenyl. = Phenylalanine; ALT = alanine aminotransferase; ALP = alka-line phosphatase; GGT = gamma-glutamyl transferase; NEFA = non-esterified fatty acids; A/G ratio = albumin/globulin (A/G) ratio; BUN = blood urea nitrogen; Cholest. = cholesterol; T.Protein = total protein; TAG = Tri-glycerides. * Loadings with an absolute value exceeding 0.4 in either direction.

**Table 2:** Principal components (PC), main explanatory biomarkers, eigenvalues (λ), variance, and cumulative sum of the explained variance based on Principal Components Analysis (PCA) performed on each of three groups of biomarkers in 440 Holstein dairy cows.

Principal Components within each model ^1^	Main explanatory biomarkers ^2^	Eigenvalue (λ) ^3^	Variance ^4^	Cumulative variance ^4^
*Amino acids intermediate of glucogenic pathways*				
g1	Serine	9.3	0.54	0.54
g2	Aspartic acid, Glutamic acid	1.8	0.10	0.65
g3	Tryptophan, Phenylalanine	1.4	0.08	0.74
*Amino acids intermediate of ketogenic pathways*				
k1	Lysine, Leucine, Isoleucine, Tryptophan	4.2	0.71	0.71
*Blood chemistry analytes*				
a1	Citrulline, Ornithine, BUN	1.8	0.20	0.20
a2	Bilirubin, NEFA	1.4	0.12	0.32
a3	A/G ratio, Total protein, GGT	1.2	0.10	0.42
a4	Taurine, Glucose	1.1	0.08	0.50
a5	Alkaline Phosphatase, GGT	1.0	0.08	0.58
a6	Cholesterol, Albumin	1.0	0.07	0.65

^1^ Nominal variable created to identify each PC. The letter ‘g’ refers to glucogenic, ‘k’ to ketogenic, and ‘a’ to analytes. The numbers refer to the hierarchical order within each group based on their respective eigenvalue.

^2^ Biomarkers that explain most of a given principal component. Abbreviations: BUN = blood urea nitrogen; NEFA = non-esterified fatty acids; A/G ratio = albumin/globulin ratio; GGT = gamma-glutamyl transferase.

^3^ Eigenvalue is a measure of the data’s covariance. The order of the eigenvalues (e.g., highest to lowest) provides a rank of significance of the principal components.

^4^ Proportion of variance and cumulative variance from 0 to 1, for each individual principal component. Values are relative to each group. E.g.: PC’s 1 to 3 account for 74% of the variance within the glucogenic pathway group, or PC number four alone account for 71% of the variance within the ketogenic pathway group.

### PCA—multivariable logistic regression analysis

Based on the multivariable logistic model for HYK+ wk1 (Table 3), cows with elevated PC a2 (led by bilirubin and NEFA concentrations) in the dry period were 29% more likely to be HYK+ wk1 than non-HYK (OR: 1.29, 95%CI: 1.02–1.68; *P* = 0.04). Similarly, cows were 2.77 times more likely to be HYK+ wk1 when having elevated PC a5 (led by ALP and GGT concentrations) in the dry period than non-HYK (OR: 2.77, 95%CI: 1.61–4.97; *P* <0.001).

**Table 3:** Estimates of odds ratio (OR; 95% CI), and *P*-value, from logistic regression models used to assess the association between principal components (PC; based on metabolites measured at 21 days prepartum) and the likelihood of having hyperketonemia only during the first week postpartum compared with cows without hyperketonemia in 332 Holstein dairy cows.

	Univariable		Multivariable	
PC ^1^	OR (95%CI)	*P*-value	OR (95%CI)	*P*-value
*Amino acids intermediate of glucogenic pathways*				
g1	0.89 (0.77–1.02)	0.11	0.92 (0.78–1.08)	0.35
g2	1.02 (0.78–1.30)	0.84	–	–
g3	1.12 (0.93–1.37)	0.22	–	–
*Amino acids intermediate of ketogenic pathways*				
k1	0.90 (0.74–1.07)	0.27	–	–
*Blood chemistry analytes*				
a1	0.90 (0.70–1.16)	0.46	–	–
a2	1.26 (0.96–1.61)	0.07	1.29 (1.02–1.68)	0.04
a3	1.17 (0.91–1.47)	0.17	0.67 (0.40–1.16)	0.14
a4	1.39 (1.07–1.80)	0.01	1.28 (0.91–1.82)	0.15
a5	3.44 (2.11–5.93)	< .001	2.77 (1.61–4.97)	< .001
a6	1.24 (0.94–1.71)	0.15	1.30 (0.94–1.77)	0.08

Hyperketonemia status: HYK positive cows only during wk1 (n = 31); HYK negative cows during the first two weeks of lactation (n = 301). Multivariable models included PC from the univariable models with a P ≤ 0.20.

^1^ Principal components (PC) are mainly explained by the following biomarkers, g1: Serine; g2: Aspartic acid, Glutamic acid; g3: Tryptophan, Phenylalanine; k1: Lysine, Leucine, Isoleucine, Tryptophan; a1: Citrulline, Ornithine, blood urea nitrogen; a2: Bilirubin, non-esterified fatty acids; a3: albumin/globulin ratio, Total protein, gamma-glutamyl transferase; a4: Taurine, Glucose; a5: Alkaline Phosphatase, gamma-glutamyl transferase; a6: Cholesterol, Albumin.

No association was observed between PCs based on metabolites measured during the dry period and HYK+ wk2 as compared with non-HYK cows (Table 4).

**Table 4:** Estimates of odds ratio (OR; 95% CI), and *P*-value, from logistic regression models used to assess the association between principal components (PC; based on metabolites measured at 21 days prepartum) and the likelihood of having hyperketonemia only during the second week postpartum compared with cows without hyperketonemia in 327 Holstein dairy cows.

	Univariable		Multivariable	
PC ^1^	OR (95%CI)	*P*-value	OR (95%CI)	*P*-value
*Amino acids intermediate of glucogenic pathways*				
g1	0.99 (0.85–1.15)	0.92	–	–
g2	0.88 (0.64–1.17)	0.43	–	–
g3	1.10 (0.89–1.37)	0.34	–	–
*Amino acids intermediate of ketogenic pathways*				
k1	0.93 (0.76–1.13)	0.51	–	–
*Blood chemistry analytes*				
a1	0.99 (0.75–1.30)	0.97	–	–
a2	0.13 (0.81–1.51)	0.41	–	–
a3	1.23 (0.97–1.57)	0.06	1.09 (0.66–1.53)	0.68
a4	0.98 (0.69–1.35)	0.92	–	–
a5	1.76 (1.10–2.88)	0.02	1.10 (0.71–1.92)	0.71
a6	1.24 (0.94–1.71)	0.15	1.30 (0.94–1.77)	0.08

Hyperketonemia status: HYK positive cows only during wk2 (n = 26) and HYK negative cows during the first two weeks of lactation (n = 301). Multivariable models included PC from the univariable models with a P ≤ 0.20.

^1^ Principal components (PC) are mainly explained by the following biomarkers, g1: Serine; g2: Aspartic acid, Glutamic acid; g3: Tryptophan, Phenylalanine; k1: Lysine, Leucine, Isoleucine, Tryptophan; a1: Citrulline, Ornithine, blood urea nitrogen; a2: Bilirubin, non-esterified fatty acids; a3: albumin/globulin ratio, Total protein, gamma-glutamyl transferase; a4: Taurine, Glucose; a5: Alkaline Phosphatase, gamma-glutamyl transferase; a6: Cholesterol, Albumin.

The univariable logistic regression analyses comparing PCs in HYK+ wk1 vs. HYK+ wk2 (i.e., model 3) identified a5 as the sole PC with *P* ≤ 0.20 (Table 5). Based on the final multivariable logistic model, cows with elevated PC a5 (led by ALP and GGT) in the dry period were 3.18 times more likely to be HYK+ wk1 than HYK+ wk2 (OR: 3.18, 95%CI: 1.34–8.73; *P* = 0.013).

**Table 5:** Estimates of odds ratio (OR; 95% CI), and *P*-value, from logistic regression models used to assess the association between principal components (PC; based on metabolites measured at 21 days prepartum) and the likelihood of having hyperketonemia only during the first week postpartum compared with cows with hyperketonemia only during the second week postpartum in 57 Holstein dairy cows.

		Univariable		Multivariable	
PC ^1^	Description	OR (95%CI)	*P*-value	OR (95%CI)	*P*-value
*Amino acids intermediate of glucogenic pathways*					
g1	Glucogenic	0.87 (0.69–1.08)	0.23	–	–
g2	Glucogenic	1.19 (0.79–1.91)	0.40	–	–
g3	Glucogenic	1.03 (0.74–1.43)	0.84	–	–
*Amino acids intermediate of ketogenic pathways*					
k1	Ketogenic	0.94 (0.70–1.27)	0.72	–	–
*Blood chemistry analytes*					
a1	Analytes	0.90 (0.61–1.32)	0.59	–	–
a2	Analytes	1.09 (0.78–1.58)	0.58	–	–
a3	Analytes	0.77 (0.39–1.43)	0.42	–	–
a4	Analytes	1.64 (1.01–2.92)	0.06	–	–
a5	Analytes	2.74 (1.21–6.99)	0.02	3.18 (1.34–8.73)	0.01
a6	Analytes	1.18 (0.84–1.80)	0.35	–	–

Hyperketonemia status: HYK positive cows only during wk1 (n = 31) and HYK positive cows only during wk2 (n = 26). Multivariable models included PC from the univariable models with a P ≤ 0.20.

^1^ Principal components (PC) are mainly explained by the following biomarkers, g1: Serine; g2: Aspartic acid, Glutamic acid; g3: Tryptophan, Phenylalanine; k1: Lysine, Leucine, Isoleucine, Tryptophan; a1: Citrulline, Ornithine, blood urea nitrogen; a2: Bilirubin, non-esterified fatty acids; a3: albumin/globulin ratio, Total protein, gamma-glutamyl transferase; a4: Taurine, Glucose; a5: Alkaline Phosphatase, gamma-glutamyl transferase; a6: Cholesterol, Albumin.

The univariable logistic regression analyses comparing PCs in HYK+ wk1 vs. HYK+ wk2 (i.e., model 3) identified a5 as the sole PC with *P* ≤ 0.20 (Table 5). Based on the final multivariable logistic model, cows with elevated PC a5 (led by ALP and GGT) in the dry period were 3.18 times more likely to be HYK+ wk1 than HYK+ wk2 (OR: 3.18, 95%CI: 1.34–8.73; *P* = 0.013).

The PCs that were significantly associated with the likelihood of having HYK during the first two consecutive weeks postpartum as compared with cows HYK- during the same period (Table 6) were g1 (led by Serine), g3 (led by Tryptophan, Phenylalanine), k1(led by Lysine, Leucine, Isoleucine, Tryptophan), a2 (led by bilirubin and NEFA), a5 (led by ALP and GGT).

**Table 6:** Estimates of odds ratio (OR; 95% confidence interval), and *P*-value, from logistic regression models used to assess the association between principal components (PC; based on metabolites measured at 21 days prepartum) and the likelihood of having hyperketonemia during the first two consecutive weeks postpartum compared with cows without hyperketonemia during the same period in 325 Holstein dairy cows.

	Univariable		Multivariable	
PC ^1^	OR (95%CI)	*P*-value	OR (95%CI)	*P*-value
*Amino acids intermediate of glucogenic pathways*				
g1	0.88 (0.74–1.03)	0.13	1.50 (1.08–2.15)	0.01
g2	0.75 (0.53–1.04)	0.11	1.15 (0.76–1.69)	0.46
g3	1.50 (1.20–1.91)	< .001	2.49 (1.63–4.06)	0.001
*Amino acids intermediate of ketogenic pathways*				
k1	0.75 (0.60–0.94)	0.01	0.67 (0.51–0.84)	0.001
*Blood chemistry analytes*				
a1	0.83 (0.62–1.10)	0.22	–	–
a2	1.36 (1.08–1.75)	0.01	1.43 (1.12–1.93)	0.01
a3	1.23 (0.95–1.58)	0.07	0.83 (0.43–1.60)	0.59
a4	0.96 (0.67–1.33)	0.82	–	–
a5	2.84 (1.70–5.00)	< .001	1.92 (1.22–3.07)	0.01
a6	1.34 (0.95–1.95)	0.10	1.11 (0.78–1.66)	0.54

Hyperketonemia status: HYK positive cows only during wk1 (n = 24) and HYK negative cows during the first two weeks of lactation (n = 301). Multivariable models included PC from the univariable models with a P ≤ 0.20.

^1^ Principal components (PC) are mainly explained by the following biomarkers, g1: Serine; g2: Aspartic acid, Glutamic acid; g3: Tryptophan, Phenylalanine; k1: Lysine, Leucine, Isoleucine, Tryptophan; a1: Citrulline, Ornithine, blood urea nitrogen; a2: Bilirubin, non-esterified fatty acids; a3: albumin/globulin ratio, Total protein, gamma-glutamyl transferase; a4: Taurine, Glucose; a5: Alkaline Phosphatase, gamma-glutamyl transferase; a6: Cholesterol, Albumin.

## Discussion

In this cohort study, we profiled plasma metabolites of dairy cows during the late dry period and characterized their correlations. In addition, we specified biomarkers associated with the occurrence of HYK at different intervals postpartum based on PCA. To our knowledge, there are no previous studies evaluating the reasons why HYK occurring at different intervals postpartum may have different consequences on the health and productivity of dairy cows.

The results of this study highlight the complex correlation between metabolites and the potential role of specific metabolites in the development of HYK in early lactation that can guide further research on the control of the disease.

The strong correlation among AAs observed in our study reflects their mutually constrained relationship likely imposed by their physical and chemical properties [40]. Simultaneously, these AAs were strongly negatively correlated with NEFA, which may be explained by the role of AAs in the energy balance. In ruminants, AAs obtained from protein catabolism and absorption, are one of the major substrates for the gluconeogenesis process together with propionate, lactate, and glycerol [41, 42]. When these substrates are scarce, as usually occurs during periods of nutrient deficit as the transition period, there is a rise in the concentration of circulating fatty acids mobilized from the adipose tissue [4, 43]. This is further supported by our results, in which we observed that NEFA was strongly negatively correlated with most AAs (e.g., alanine, methionine, tyrosine). In addition, bilirubin was strongly negatively correlated with AAs. The high concentrations of bilirubin and NEFA may be related as the increase of bilirubin concentration, usually observed in dairy cows around parturition, has been attributed to the diminished hepatic uptake of bilirubin in favor of NEFA [44, 45]. Moreover, the increase of bilirubin observed during the dry period may suggest degeneration of liver cells. Together with GGT, bilirubin concentrations in blood is a surrogate marker of the proper function of the liver; thus, it is one of the metabolites frequently assessed to determine liver function because it is released into the bloodstream when liver cell damage occurs [46].

As reported in the current study, the dataset consists of 36 metabolites with a high degree of correlation. Thus, the analysis of the dataset in its current state represents a challenge due to the loss of valuable data in the process and potentially biased results. For instance, only 57 cows are available to evaluate the associations between metabolites-based PCs in the dry period in cows with HYK in the first vs. the second week of lactation (Table 5). The PCA is a data reduction technique that can be used to reduce multivariable datasets while minimizing information losses before being analyzed by conventional techniques such as regression [31, 47]. However, the downside of implementing PCA on the dataset, is that principal components are not as readable and interpretable as original variables. We performed the PCA separately on three metabolite-containing groups according to their metabolic pathways. This approach has been previously used to facilitate a logical interpretation of the components when evaluating their association with certain outcomes (i.e., the occurrence of HYK) due to the clear distinction between each group [34–36]. However, clustering metabolites in different groups prevented us from observing the potential interactions between different groups which is a limitation of the study.

Our study showed an association between both PCs a2 (led by bilirubin and NEFA) and a5 (led by ALP and GGT) measured 21 days before calving and HYK only in the first week of lactation as compared with non-HYK cows. In addition, a5 (i.e., ALP and GGT) remained significantly associated with HYK+ wk1 as compared to HYK+ wk2. Both ALP and GGT, the leading metabolites of g5, suggest the presence of liver dysfunction during the transition period in cows HYK+ in the first week of lactation given the relationship between bilirubin, GGT, and ALP with liver function [46].

Bilirubin is a subproduct of the enzymatic degradation of the hemolysis process and its clearance is dependent on liver enzymes. When the liver is injured, the clearance of bilirubin declines, being indicative of liver dysfunction [48]. Meanwhile, GGT and ALP are enzymes that accomplish a variety of functions in the body such as protein transport and cell growth [49]. These enzymes are released into the bloodstream when damage occurs in cells of the liver, skeletal muscle, or heart [50]. Therefore, the increase of principal components led by those metabolites suggests that some level of liver damage may be present at 3 weeks prepartum in cows HYK+ wk1 compared with cows HYK+ wk2.

The indication of liver damage prepartum in cows HYK+ wk1 unlike HYK+ wk2 may be one of the reasons for the different association between HYK occurring at different intervals postpartum and health and productive outcomes throughout the lactation in dairy cows [6, 11, 13]. Moreover, liver damage could be related to fatty liver syndrome as further supported by the identification of NEFA as another specific biomarker of HYK in wk1. During the late dry period, the energy deficit triggers an excessive mobilization of NEFA [4]. The increased blood NEFA concentration leads to more NEFA taken up by the liver exceeding its complete oxidation capacity. As a consequence fatty acids in the form of triglycerides accumulates excessively in the hepatocytes, contributing to hepatocyte degeneration [4, 51, 52]. Both, the overwhelmed capacity of the liver to oxidize fatty acids and the further hepatocyte degeneration lead to a further increase in BHB concentration in blood [5]. Our findings are in agreement with results from a recent study, in which researchers using a PCA approach to study changes in metabolic profiles of dairy cows reported an increase in principal components led by NEFA and AST during the dry period attributed to energy deficiency and liver dysfunction [34]. In our study, none of the PCs led by biomarkers characteristic of liver dysfunction and fatty liver were associated with HYK+ wk2.

Inflammation during the transition period is also an element that is linked with the transition performance and is likely to play a role in the negative health and productivity of cows with HYK+ wk1. In a recent review, Horst et. al. (2021) [2], discussed how all major transition cow diseases and disorders (i.e., metritis, mastitis, ketosis, milk fever, and retained placenta) are preceded by a heightened inflammatory response. In this review, the authors speculate that this increased inflammatory response is likely linked to a compromised epithelial barrier at the uterus, mammary gland, and/or the gastrointestinal tract. Although infections tend to occur close to or immediately after parturition, other events occurring before parturition such as colostrogenesis and remodeling in the udder or the dilation of uterine structures are also sources of inflammation [2, 53]. During inflammation an increase of reactive oxygen species leads to a state of oxidative stress interfering with the transport of glucose, causing a state of insulin resistance in the liver and peripheral tissues [54–56]. Thus, it is likely that a different inflammation process and response during the transition period may be reflected in the levels of BHB in early lactation.

We speculate that the reason for elevated BHB concentration at wk2 postpartum may be more related to the elevated demands of glucose in early lactation required by milk production instead of liver injury and inflammatory response. Specifically, the increased lipolysis postpartum, and consequently elevated blood BHB concentration, in these cows is likely a response to the mismatch between feed intake and nutrient requirements to support lactogenesis [57–59]. It has been reported that cows in which the demands of glucose exceed the gluconeogenic capacity of the liver develop HYK later in lactation (i.e., 3 to 6 weeks postpartum) [5, 60]. Thus, an elevated pressure for milk yield shortly after calving that is not matched by an increased feed intake may have been occurring earlier in the lactation resulting in HYK as early as the second week postpartum. Different hormones and molecules mediate the control of the adaptive response during this period, some of them tightly related, such as insulin and glucose. In an anabolic state, insulin will induce lipogenesis and inhibit lipolysis in the adipose tissue [61]. A decreased response to insulin (i.e., insulin resistance) affects the use of glucose and thus plays a fundamental role in the physiological adaptation of dairy cows [62, 63]. As these cows may not necessarily be affected by liver dysfunction but a transient energy shortage, it could be a reason to explain why they do not experience negative health and productive effects throughout the lactation as cows with HYK in the first week postpartum [10].

The evaluation of the association between PC and the likelihood of having HYK during the first two consecutive weeks postpartum resulted in associations across the three metabolic pathways. Then, suggesting that cows with HYK in both weeks of lactation had a general metabolic disbalance at the beginning of the late dry period rather than only related to liver dysfunction as cows HYK+ wk1. For instance, k1, the only PC related to a ketogenic pathway and led by lysine, leucine, isoleucine, and tryptophan (in this order) was negatively associated with the likelihood of HYK during the first two consecutive weeks postpartum. During the late dry period, there is a negative protein balance due to increasing demand by the mammary tissues and conceptus growth, which is considered of great importance [64]. Moreover, limited intake and low duodenal availability of indispensable AA during the periparturient period have been associated with poor immune response, tissue function, and proliferation of visceral and liver tissues [64–66]. In a recent study, Fehlberg et al. (2020) [67] reported that cows fed with a diet containing rumen-protected lysine from -28 days to calving had lower BHB concentrations during the first week postpartum as compared with cows fed with a diet without rumen-protected lysine during the same period. This effect has also been observed in other studies [68, 69]. Similarly, reduced concentrations of lysine, leucine, isoleucine, and tryptophan have been reported in cows with clinical ketosis postpartum [70]. The glucogenic-related PCs g1 (led by Serine) and g3 (led by tryptophan and phenylalanine) were also associated with the development of HYK during the first two consecutive weeks of lactation. An abnormal tryptophan metabolism has been reported to contribute to the pathogenesis of ketosis [71]. Tryptophan is an essential amino acid that participates in the regulation of inflammation and insulin resistance [72, 73].

In our study, we used 1.2 mmol/L as our threshold to declare HYK. Other studies have used different thresholds, ranging from 1.0 to 1.4 mmol/L to define HYK [11, 21, 74]. Different concentrations have been associated with subsequent negative health, productive and reproductive events such as mastitis, milk drop, or reproductive failure [6, 7, 11]. Therefore, the hypothesis generated by this work apply when defining HYK based on a threshold of 1.2 mmol/L. Ideally, blood samples would be collected twice a week for all cows to account for the 5 days that on average BHB concentration remains over the threshold of 1.2 mmol/L in blood [7]. In our study, as samples taken at 7 ± 2 DIM had equal chance to fall before or after day 7 a a potential misclassification bias would be non-differential. In other words, a potential bias due to sampling schedule would be conservative allowing us to make an inference about the link between biomarkers of liver dysfunction and HYK postpartum. Still further research is needed to investigate the hypothesis generated by this work and to understand the underlying mechanisms associated with the different metabolic adaptations in the peripartum of cows that develop HYK during week 1 or week 2 of lactation.

### Conclusion

Our exploratory results based on PCA, suggest a potential association between biomarkers related to liver dysfunction and fatty liver (i.e., bilirubin, NEFA, alkaline phosphatase [ALP], and gamma-glutamyl transferase [GGT]) measured at three weeks before parturition and HYK in the first week of lactation. Meanwhile, no evidence for an association was observed between the biomarkers measured during the dry period and HYK occurring during the second week of lactation. In addition, results suggest that cows with HYK in both of the first two weeks of lactation had an overall metabolic disbalance during the onset of the late dry period, which based on PCs, encompass biomarkers related to glucogenic and ketogenic metabolic pathways as well as liver dysfunction and fatty liver.

## Supporting information

S1 Data (CSV)
